# Physicochemical Properties and Consumer Appeal of High Pressure Structured Pea and Chickpea Isolate-Enriched Whole Concord Grape Gels

**DOI:** 10.3390/gels11120972

**Published:** 2025-12-02

**Authors:** Viral Shukla, Yichen Yang, Olga I. Padilla-Zakour

**Affiliations:** Cornell AgriTech, Department of Food Science, Cornell University, Geneva, NY 14856, USA; vs354@cornell.edu (V.S.); yy2264@cornell.edu (Y.Y.)

**Keywords:** high pressure processing, quality, protein, sensory, consumer appeal

## Abstract

Protein-enriched fruit gels, such as spoonable sauces and cuttable gels, can meet consumers’ desire for high protein/fiber value-added health foods. High pressure processing (HPP) is a nonthermal pasteurizing method that has shown additional usage as a novel structuring method for gels by affecting protein–protein interactions. This work studied HPP (575 MPa, 3 min, 5 °C) compared to heat (85–90 °C, 3–10 min) pasteurization as a method to produce novel fruit gels from whole Concord grapes enriched with 4, 6, and 8% (*w*/*w*) chickpea and pea protein. Physicochemical and rheological analyses were conducted, as well as sensory evaluation of a model gel. Heat-treated gels produced spoonable high viscosity gels compared to free standing gels produced through HPP. Chickpea protein-enriched samples exhibited a greater change with an increase in heat processing due to non-protein constituents compared to pea protein. Sensory analysis showed a desire for added nutritional value, though flavor was ultimately the deciding factor in preference, with heat-treated gels achieving higher liking scores compared to a HPP counterpart.

## 1. Introduction

Fruit purees and gels, such as apple sauce and cranberry sauce, are a typical, convenient form of fruit consumption. Concord grapes (*Vitis labrusca*) have been grown widely for both fresh consumption and in juices and jams, offering a rich profile of phytochemicals and nutrients that contribute to overall dietary health [1]. Concord grapes may be used to produce fruit gels; however, their characteristic large seeds may introduce grittiness into the product, but elevate the product’s nutritional value by augmenting fiber, protein, and fatty acid content [2]. Concord grapes have a high acid content compared to other fruits, allowing them to withstand the buffering capacity of added proteins, leading to opportunities in a fruit-based protein-fortified product.

Proteins isolated from peas and chickpeas are gaining attention for their nutritional benefits, hypoallergenicity, and plant-based functional properties [3,4]. Additionally, the low water and land requirements to produce these proteins can enhance the sustainability of food production by reducing the environmental impact of agriculture [5]. The addition of these plant proteins may be used to fortify grape products to produce clean-label, protein-fortified foods that fulfil consumers’ desires for higher protein and fiber content, fewer added sugars, and shorter ingredient label length [6].

High pressure processing (HPP) is a non-thermal processing method that has been reported in the literature as both a pasteurization and physical-structuring method. HPP has been shown to produce safe, phenolic-rich products through the use of non-thermal pasteurization of acidic (pH < 4.6) foods [2,7]. Beyond pasteurization, pressures in the 300–600 MPa range cause irreversible denaturation of proteins, leading to the formation of new bonds in a stabilized gel matrix [8]. A gel can be created both by heat and pressure as a denaturation force upon proteins, causing an unfolding that allows internal hydrophobic groups to create new bonds [9]. HPP can also enact gelation through gelatinization of starches; however, starch granules maintain their granule shape compared to the structure loss from heat treatment [10]. HPP has been shown to enact these functional and structural changes in pulse protein solutions, leading to gelled products [11,12]. This gelation of pulse proteins is affected by a lowered pH due to changes in protein–protein interactions near the isoelectric point [13]. While this reduction in pH is often enacted through the addition of acids, such as hydrochloric or glucono-δ-lactone, an acidic matrix such as grape puree may serve to reduce pH for both safe pasteurization and enhanced structuring. There is an opportunity to use HPP as both a microbial-stabilizing and structuring method for novel gel foods; however, viscosity and texture affect consumer acceptability and perception of flavor. Thus, understanding how HPP can affect texture can provide a basis for developing suitable gel textures [14,15].

Both conventional heat treatment and HPP are used as pasteurization methods to reduce pathogen load in acidic fruit purees. The addition of protein meets consumers’ desire for increased protein foods but also leads to opportunities for protein-processing functionality [6]. HPP was used in this study as a pasteurization method for its secondary effect on protein tertiary structures. HPP at 575 MPa forms new disulfide bonds and hydrogen bonds to stabilize the denatured state of the protein, allowing for a structured gel product [16,17]. Heat also leads to a similar denaturation of proteins, but may affect covalent bonding as well. The acidic grape puree maintains low pH with added protein, allowing for a protonated state of the exposed groups, decreasing the electrostatic repulsions, leading to enhanced aggregation [18].

This study aimed to utilize the whole Concord grape (including seeds), along with the application of pea and chickpea proteins, to create nutritious and clean-label plant-based gel products. The study analyzed the physicochemical and structural properties of the product and assessed the effects of HPP and traditional heat pasteurization as preservation and structuring methods on the quality and sensory appeal of the final product.

## 2. Results and Discussion

Minimal-ingredient protein-enriched Concord grape gels were structured using HPP and heat processing. These products were analyzed for various physicochemical and textural properties to elucidate characteristics that are important for consumer appeal. These samples were further analyzed by sensory analysis to determine consumer perception of such products and their feasibility for industry adoption.

### 2.1. Physicochemical Properties

**Figure 1:**
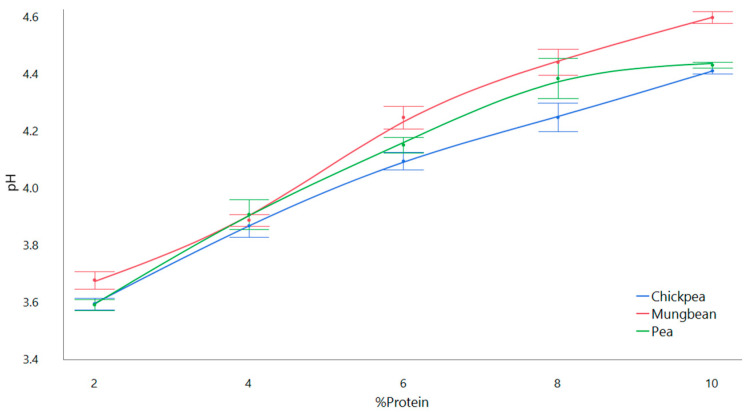
pH curve of Concord grape puree with added protein.

Controlling pH is important for ensuring the quality and safety of the final product. A final pH of 3.8–4.4 was desired to produce a gel that was microbially safe in terms of inhibition of *Clostridium botulinum* spores’ growth post-HPP. pH was correlated with %protein due to the buffering effect increasing the initial low pH provided by the grape puree (Figure 1). All mung bean and 2% and 10% pea and chickpea formulations were excluded from further study due to undesirable physicochemical and sensorial properties from internal analysis by the research group.

#### 2.1.1. Color

Color and visual appearance are primary factors that influence consumer perception and acceptance. Anthocyanins from grape skin are responsible for the purple colors observed, though their sensitivity to heat and pH may lead to potential color degradation during processing. Color is listed in Table 1. According to the color change thresholds reported, ΔE > 1.5 indicates a noticeable human perceptible change in color [19]. Generally, HPP exhibited a greater perceptible change in color compared to heat samples, with individual color component changes being consistent with those found in the literature [2]. No significant difference was found for a* values across all samples; however, addition was significant, indicating that higher levels of protein addition may lead to a significant change in redness. Values for L* followed a similar trend with protein type being the main significant factor. There was also no significant difference in b* in pea samples, though chickpea samples differed from pea samples and within themselves. Despite there being no significant difference between treatments, the ΔE values for HPP were higher than for heat, indicating a possible consumer-perceptible significant color change. ΔE and Browning Index (BI) trended positively with protein content in heated samples, indicating protein–heat interactions such as Maillard browning creating greater color change than HPP, which has minimum thermal generation.

#### 2.1.2. Viscosity

**Figure 2:**
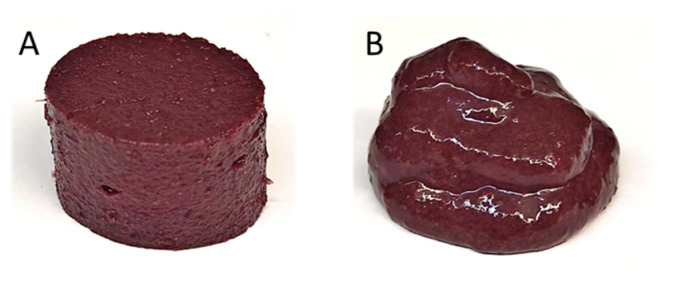
Whole Concord grape puree with added protein structured by (**A**) high pressure processing and (**B**) heat treatment.

**Figure 3:**
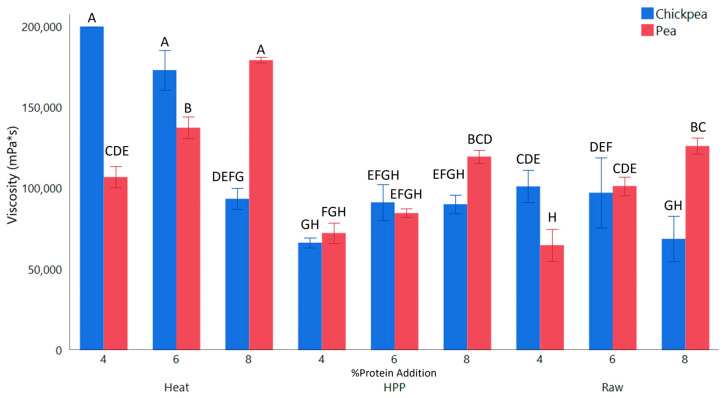
Viscosity of whole Concord grape gels with added 4, 6, and 8% pea or chickpea protein structured by HPP or heat. ^A–H^ Different letters are significantly different (*p* < 0.05).

Viscosity is an important rheological property that can be linked to gel composition as well as its macrostructure. HPP gels were free standing, while those with heat flowed similarly to a thick puree (Figure 2). For products formulated with pea protein, viscosity increased consistently with higher protein content in unprocessed samples, likely due to an increase in total solids (Figure 3). HPP sample viscosity increased with protein content, which is seen in the literature [11]. Heat treatment increased viscosity compared to HPP due to the difference in macro-gelation. HPP gel’s free standing structure would break down and move away from the continuous shear from the spindle, leading to a lower viscosity due to decreased impact with the shaft. Heat samples, on the other hand, produced a flowing gel that would not break down as easily from the mild shear of the spindle, leading to the gel flowing towards the spindle in the center; thus, a higher viscosity.

Chickpea gels exhibited an opposite trend from pea gels when processed with heat. The chickpea protein used in this study had a lower percent total protein, leading to a relatively higher amount of chickpea starch. Starch can gelatinize and disrupt the protein matrix, leading to a weaker gel [20]. Nonthermal processing does not lead to starch gelatinization to the same extent, thus this was not exhibited in the HPP samples [21]. The chickpea protein used in this study also contained a higher percentage of fat, which has been shown to increase the viscosity of gels and result in a higher G′, which may also lead to the difference seen between the two protein types [22,23].

### 2.2. Total Monomeric Anthocyanin Content (MA)

**Figure 4:**
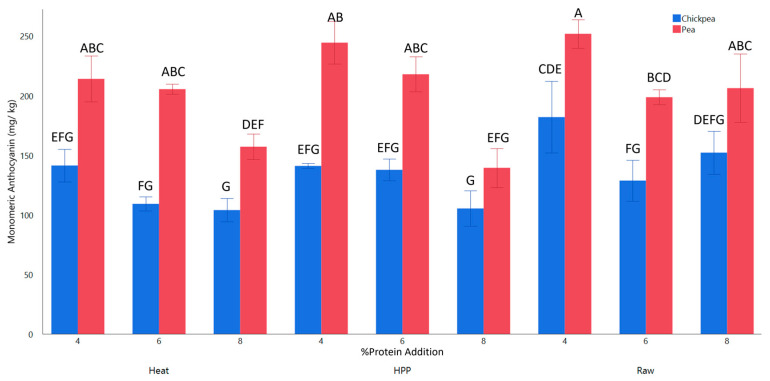
Total monomeric anthocyanin content of whole Concord grape gels with added 4, 6, and 8% pea or chickpea protein structured by HPP or heat. ^A–G^ Different letters are significantly different (*p* < 0.05).

Anthocyanin is the main red pigmented antioxidant found in red grapes such as Concord grapes. All MA values fell within the detected range for red grape varieties, which is from 40.3 mg/kg to 990.8 mg/kg fresh weight, when prepared without seeds (Figure 4) [24]. Both treatment methods led to a decrease in MA, particularly in heat samples with the highest parameters compared to nonthermal HPP. The change in color may be attributed to this decrease in MA content (Figure 2). Heat treatment reduces MA, as evidenced by studies showing that the higher temperatures used in this study accelerate the degradation of anthocyanins in blueberry and cherry juices [25,26]. It has been shown that dual HPP (400+ MPa) and heat treatment can lead to the degradation of anthocyanin extract [27]; however, HPP up to 600 MPa showed no significant difference in cyandin-3-glucoside content from the control in food matrices of bayberry juice and grape puree [2,28]. This was supported by statistical analysis indicating that HPP and the control were non-significantly different, while heat was significantly (*p* < 0.05) different.

As expected, MA was negatively correlated (*p* < 0.05) with %protein replacement, though pea samples exhibited higher amounts of anthocyanin. Both pea and chickpea proteins contain anthocyanins, though not in the amount present in grape puree [4,29]. It has been shown that non-covalent interactions between polyphenols and pea protein can lead to an enhancement of the polyphenol’s stability; given that HPP denatures proteins and increases non-covalent interactions, the high retention of MA reported in this study may be enhanced by this stabilizing effect [30,31]. Chickpea protein has also shown complexion with pectin and polyphenols for a protective effect; however, the complexity of the matrix in this study and suboptimal pH may lead to a decreased effect [32].

### 2.3. Rheological Properties

**Figure 5:**
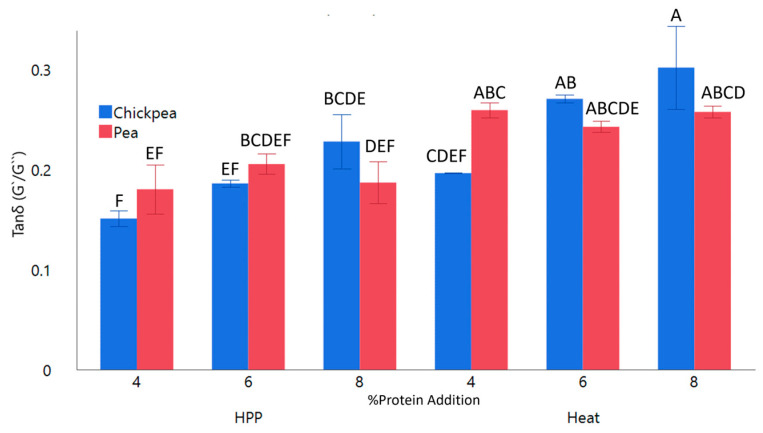
Tan δ of whole Concord grape gels with added 4, 6, and 8% pea or chickpea protein structured by HPP or heat. ^A–F^ Different letters are significantly different (*p* < 0.05).

Small amplitude oscillatory shear analysis was used to determine G′, G″, and tan δ of the gelled samples to understand their microstructuring. Overall, all samples indicated a gel-like structure compared to the fluid-like behavior based on having a tan δ < 1 (Figure 5). Despite the flowing consistency of heat-treated samples, they still exhibited a gel-like structure at the micro level. Protein type was statistically significant, though this was not the case overall for samples as interaction was non-significant. Pea protein-enriched gels maintained relatively similar tan δ values, while chickpea gels trended with %protein, likely due to their higher fat content [23]. HPP gels exhibited a significantly lower tan δ and higher G′ compared to heat-treated samples, though the interaction between process and protein was not significant. These results differed from those described by Hall and Moraru (2021) who found stronger gel-like behavior from heat-treated samples [33]. This may be due to the stronger heat treatment in that study than this one, leading to the possibility of modulating heat or HPP treatment to produce a preferred gel strength. Insoluble fiber, such as that found in grape puree, has been shown to disrupt gel structure, leading to weaker gel strength [34]. Conversely, starch has been shown to produce harder and denser gels, which may have led to a higher tan δ with higher protein substitution as %addition was statistically significant [10]. Despite stronger heat treatment in higher protein-content gels, there did not appear to be an effect on pea gels, indicating a protein-type dependency.

### 2.4. Sensory Properties

Preliminary tastings indicated that the best formulation was the gel prepared with 6% pea protein; thus, a formal sensory analysis was conducted to assess the effect of HPP vs. heat processing on acceptability. Nine-point hedonic scores between heat-treated and HPP-treated samples are shown in Table 2. Overall, heat-treated samples were preferred over HPP ones. Color was not significantly different, which is corroborated by the lack of significant difference from analytical testing (Table 1). HPP samples were significantly lower in flavor and aroma liking scores than heat samples. Overall liking was most correlated with flavor liking (0.84), then aroma liking (0.63) and texture liking (0.70). Pulse proteins have been associated with grassy flavors and off-flavors that are reduced through heat processing [35]. In this study, perceived protein flavor-amount liking was not significantly different (3.20 heat, 3.33 HPP), nor was aftertaste detection (30% heat, 29% HPP) and its acceptability (70% heat, 59% HPP), meaning protein flavor is not a contributing factor.

**Figure 6:**
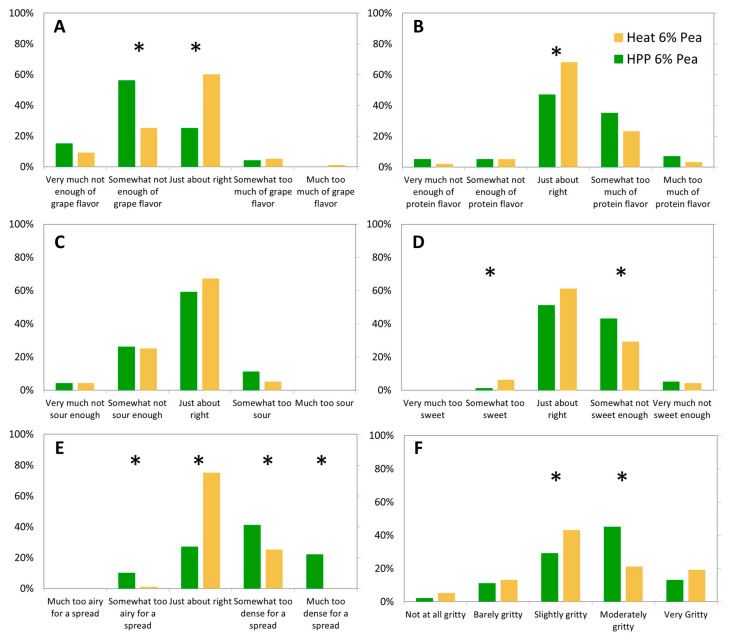
Five-point intensity and Just-About-Right results from sensory analysis: (**A**) grape flavor; (**B**) protein flavor; (**C**) sourness; (**D**) sweetness; (**E**) density; and (**F**) grittiness, of whole Concord grape gels with added 6% pea protein structured by HPP or heat. * Columns are significantly different (*p* < 0.05).

**Figure 7:**
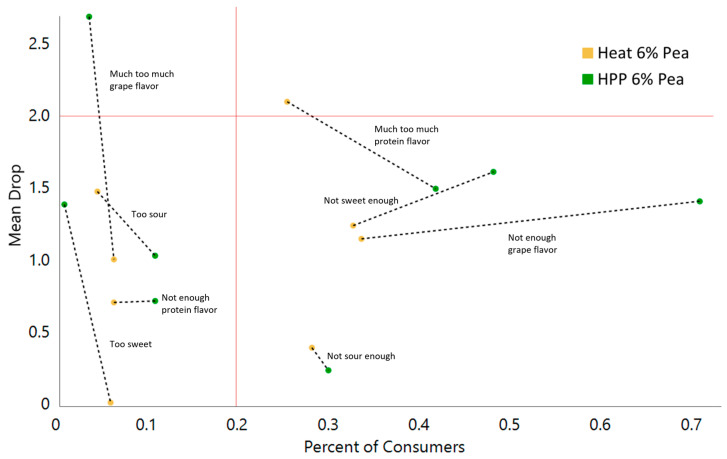
Penalty analysis from sensory analysis of 6% pea protein-enriched whole Concord grape gels structured by HPP or heat. Red lines denote critical zones.

HPP had a lower perceived grape flavor-amount liking (2.18 versus 2.65 mean values, respectively) (Figure 6). Based on penalty analysis, a higher percentage of consumers of HPP samples were sensitive to grape flavor amount, with a high %consumers reporting less-than-JAR for the grape flavor in HPP samples, but with similar effect on overall liking compared to heat (Figure 6 and Figure 7). The lower perceived flavor from the HPP gels may stem from a reduced ability to taste freestanding gels compared to flowing gels that may better coat the tongue [36].

Grape seeds provide many health benefits in terms of added fiber, fats, and phytochemicals; however, they can lead to undesired grittiness in the product. The HPP samples had non-significantly higher perceived grittiness than heat samples (3.56 and 3.37, respectively), indicating that a solid gel versus more flowing one may increase perceived gritty mouthfeel beyond that provided by the grape puree. In terms of grittiness acceptance, heat and HPP were not significantly different (67% and 59%, respectively) (Figure 7). Overall, evaluation of the model gel indicates that flavor plays an important role in consumer acceptance, followed by texture and aroma.

### 2.5. Consumer Implications

The gels enriched by pea and chickpea produced products that were self-standing and high viscosity purees, respectively. These gels fit into product categories including fruit purees (apple sauce) and fruit gels (cranberry sauce) that are suitable as snacks for children and adults. About half of the sensory correspondents indicated that a clean label was important when purchasing these types of products (Table 3). Furthermore, consumers seek products that are high in protein (55%) and fiber (53%), leading to a need for products that were produced in this study with added protein and fiber from seed-containing whole fruit. The results were in line, but higher than those reported by Mintel, in terms of protein and added sugar, indicating a higher desire for nutritive content in perceived healthy products [6]. Consumers often associate cleaner labels with naturalness and higher perceived healthfulness, supporting these findings [37,38]. Once consumers were made aware of the protein and fiber content, the lack of added sugar, and the clean label, average willingness to purchase similar pulse-enriched products increased by 0.7 points, leading to opportunities for industry to adopt added nutritional products.

It is well known that heat treatment can reduce nutritional value through the destruction of heat-labile constituents and enhanced biochemical reactions. Literature has shown that neither HPP nor mild heat (63 °C, 3 min) significantly affected Concord grape puree macro nutrition value [2]. Though heat treatment has been shown to reduce trypsin inhibitor activity, HPP-treated gels showed greater gastric proteolysis than similar heat-treated pulse protein gels despite any structural modification [39,40]. Per 140 g, the US FDA recommended amount to consume in one serving, the 6% protein gels contained almost 10 g of protein and more than 5 g of fiber (Table 4). Overall, these products meet consumers’ expectations for value-added fruit-based snacks with only three ingredients.

## 3. Conclusions

This work has shown the use of high pressure processing and heat treatment as pasteurizing and structuring treatments for pea and chickpea-enriched whole Concord grape gels. This research points to the creation of both free standing (from HPP) and flowing (from heat) gels. HPP produced harder gels that would break down by shear and had higher tan δ, while heat produced a spoonable texture that exhibited higher viscosity that could be modulated by time and temperature. Chickpea-enriched gels produced a more process-dependent product, likely due to higher starch and fat content. Gels produced by HPP were shown to have lower sensory acceptability than heat-processed samples due to lower flavor liking. Gels produced in this study contained 10 g of protein and 5 g of fiber, with consumer analysis indicating preference for value-added products such as those produced in this study. Further analysis on group-specific preference should be conducted to better understand how nutrient content and sensorial aspects are received by consumers. This work informs industry on the creation of clean-label fruit products enriched with protein from pulses and fiber from grape seed, with increased nutrition content desired by consumers. It lays the groundwork for future work around shelf-life analysis and feasibility of creating such products for the marketplace at the industrial scale.

## 4. Materials and Methods

### 4.1. Materials

Fresh Concord grapes (*Vitis labrusca* L.) were picked locally from a vineyard (Penn Yan, NY, USA) and processed at the Cornell Food Venture Center Pilot Plant (Geneva, NY, USA). Grapes were stored in a refrigerated room at 4 ± 1 °C until processing. Grapes were destemmed by hand and then ground using a pilot scale food processor (STEPHAN Microcut MC15, Riga, Latvia). Samples were stored frozen until use. Pea, chickpea, and mung bean protein isolate were provided by Shandong Jianyuan Bioengineering Co., Ltd. (Zhaoyuan City, China). Isolates had ≥85% protein content (*w*/*w*) and moisture ≤ 10%, passing an 80-mesh sieve ≥ 90%. Pectin was from Ingredion (Westchester, IL, USA). Methanol, hydrochloric acid, potassium chloride, and sodium acetate were from VWR (Radner, PA, USA).

### 4.2. Gel Preparation

An initial pH curve was produced to determine change in pH by addition of protein. Puree was mixed at 5 protein concentrations (2%, 4%, 6%, 8%, 10% *w*/*w*) using pea protein, chickpea protein, and mung bean protein, and measured for pH. All mung bean and 2% and 10% pea and chickpea formulations were excluded from further study due to undesirable physicochemical and sensorial properties from internal analysis by the research group.

**Figure 8:**
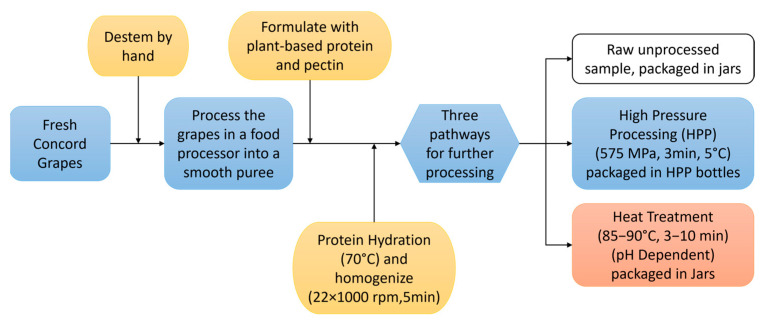
Flow diagram for the production of whole Concord grape puree and the formulated protein-enriched products.

Formulations with pea protein and chickpea protein of concentrations of 4, 6, and 8% were stabilized with 2 processing methods (heat and HPP) for further analysis. Figure 8 shows the product processing procedure followed to produce the final samples. Protein and 1% pectin were added to the prepared grape puree and heated to 70 °C and homogenized using a high-speed homogenizer at 22 × 1000 rpm for 5 min for proper hydration (HSM-100LSK, Charles Ross & Son Company, Hauppauge, NY, USA). Following homogenization, the mixture was divided into control, heat treatment, and HPP.

HPP samples were processed at the Cornell HPP Validation Center (Cornell AgriTech, Geneva, NY, USA) following biosafety level 2 guidelines, which prohibit sensory analysis testing. HPP-compatible PET bottles (4 oz, Merrimack Valley Plastics, Methuen, MA, USA) filled with to the top to avoid headspace (120 g) were packed into PET bags and vacuum sealed. Packages were loaded into a 55 L commercial high pressure processing unit (Hiperbaric 55, Hiperbaric, Burgos, Spain), using 5 °C water to transmit pressure. Product samples were pasteurized at 575 MPa for 3 min at 5 °C, parameters commonly used in the food industry to achieve a greater than 5-log reduction in relevant pathogens in acid/acidified juices/beverages (pH < 4.5). This produced a solid gel with no discernable syneresis post-processing.

For heat treatment, products were heated in a kettle, hot filled into glass jars, and then hot held in an oven based on pH. For pH 3.5–4.0, samples were heated to 85 °C then held for 3 min; for pH 4.0–4.2, samples were heated to 90 °C then held for 6 min. For pH 4.2–4.4, samples were heated to 90 °C then held for 10 min. Samples were rapidly cooled after hold time to maintain quality. This produced a soft flowing gel with no discernable serum separation post-processing.

All samples were held at 4 °C to maintain quality.

### 4.3. Physicochemical Properties

pH was measured at room temperature (22 °C) using a pH meter (OrionTM 3-star, Fisher Scientific, Waltham, MA, USA). Viscosity was measured using a ViscoQC 300 Viscometer (Anton Paar USA, Inc., Ashland, VA, USA) with a L-4 spindle. Samples were equilibrated to room temperature (22 °C) in their original bottles before measuring at 3 rpm at a steady state at 60 s; care was taken to ensure the walls of the container did not affect shearing of the gels. Viscosity was reported as mPa·s. Gel color metrics were determined using a Labscan XE colorimeter CIELAB (Hunter Associates Laboratory, Inc., Reston, VA, USA) in reflection mode. Samples were measured in a 10 mm path-length quartz cuvette. The ΔE values for absolute color difference in a sample were calculated according to Equation (1) as shown below, where L_0_, a_0_, and b_0_ are the color measurements of unprocessed control samples, and L, a, and b are the color measurements of HPP- or heat-treated samples.

(1) $$\Delta E=\sqrt{{\left(L-L_{0}\right)}^{2}+{\left(a-a_{0}\right)}^{2}{+\left(b-b_{0}\right)}^{2}}$$

The browning index (BI), which represents the purity of the brown color and is reported as an important parameter in processes where enzymatic or nonenzymatic browning takes place, was analyzed and calculated using Equation (2) [43].

(2) $$\begin{array}{c} \mathrm{BI}=\frac{100\times \left(x-0.31\right)}{0.172}\; \end{array}$$

where $x\;=\;\frac{a+1.75\times L}{5.645\times L+a-3.012\times b}.$

### 4.4. Monomeric Anthocyanin Content

For total monomeric anthocyanin content, the extraction procedure was based on the methods reported by Iland et al. and Jensen et al. with some modifications [44,45]. Briefly, the sample was mixed with acidified methanol (1% HCl, *v*/*v*) at a 1:10 ratio (*w*/*v*). After vortexing the mixture for 1 min, tubes were incubated at 40 °C for 30 min. The supernatant was transferred into new vials after centrifugation (12,000× *g*, 5 min). The supernatant was then used as an anthocyanin solution for future determination. Sample weight (M_p_) and supernatant volume (V_s_) were recorded for calculation.

Total monomeric anthocyanin content was determined using the pH adjusted method [1]. Briefly, 1 mL extracts were separately diluted with 3 mL pH = 1.0 (0.025 M, potassium chloride) and pH = 4.5 (0.4 M, sodium acetate) buffers. The mixture was gently vortexed and equilibrated at room temperature for 20 min. Deionized water was used as a blank. Absorbance readings were taken at both 520 nm and 700 nm using a UV-visible spectrophotometer (Genesys, ThermoFisher Scientific, Waltham, MA, USA). Results were calculated using the following Equation and expressed as cyanidin-3-glucoside equivalent (CGE):

(3) $$CGE\left(\frac{mg}{kg}\right)=\frac{A\times M_{w}\times DF\times 10^{-3}\times V_{s}}{\varepsilon \times L\times M_{p}}$$

where A = (A520 nm − A700 nm)pH1.0 − (A520 nm − A700 nm)pH4.5; M_w_ (molecular weight) of cyd-3-glu = 449.2 g/mol; DF (dilution factor) = 3; ε is the molar extinction coefficient = 26,900 L^−1^cm^−1^mol^−1^ for cyd-3-glu; L (pathlength) = 1 cm; Vs is the gel extraction volume (mL) and 10^−3^ is the conversion of mL to L; and M_p_ is the fresh puree weight (g) used for extraction. Results were calculated and expressed as mg CGE/kg of sample.

### 4.5. Rheological Analysis

Rheological properties were analyzed through small amplitude oscillatory shear (SAOS) analysis using an ARES strain-controlled rheometer (TA Instruments, New Castle, DE, USA). Gels were cut into a 25 mm diameter/2 mm height circle with a circular cutter and loaded on a 25 mm plate with a 2 mm interplate gap and enclosed in an isothermal chamber at 4 °C. Prior to measurement, gels were allowed to relax for 60 s in the chamber. Dynamic strain sweeps from 0.05 to 3% were conducted at a frequency of 1 rad/s to select a strain value within the linear viscoelastic region. Frequency sweeps were conducted from 1 to 100 rad/s at the selected strain value to determine storage modulus (G′), loss modulus (G″), and tan δ (G″/G′).

### 4.6. Sensory Analysis

A sensory evaluation was conducted with the 6% pea protein formulation treated with heat or HPP as the model gel. Testing was conducted at the Cornell University Sensory Evaluation Center (Ithaca, NY, USA) and approved by the Cornell University Institutional Review Board for Human Participants, protocol #1405004676, reviewed in 2021. A total of 110 consumers (73% Female, 26% male, 1% non-conforming) with normal senses of smell and taste were recruited for sensory testing from the local campus community. Informed consent forms were provided to panelists, and they received financial compensation for their participation in the study.

HPP samples were produced at an industrial HPP co-manufacturer at the same specifications (LiDestri Foods, Rochester, NY, USA). Panelists were served chilled 30 g servings in a balanced randomized block test, using 3-digit blinding codes. Samples were tested to ensure safety. The surveys were designed and conducted using RedJade Sensory Evaluation Software 6 (Curion, Deerfield, IL, USA). The affective test comprising 9-point hedonic scale questions (1—Dislike extremely to 9—Like extremely) to evaluate appearance, color, aroma, texture, flavor, and overall liking. Five-point intensity and Just-About-Right (JAR) questions were asked to evaluate sweetness, sourness, grape flavor, protein flavor, mouthfeel, consistency, grittiness, and color intensity. Panelists were also asked how well the product met their expectations (5-point) and to select which sample they preferred. A 5-point purchase intent question (1—Definitely would not purchase to 5—Definitely would purchase) was asked. Post-tasting, a final purchase intent question was asked after informing the panelists of protein and fiber content, and the ingredient list. A penalty analysis was conducted to determine mean drop on overall liking versus %consumers to elucidate differences in JAR questions between processing methods [14].

### 4.7. Statistical Analysis

All results are presented as mean values of the data from experiments performed in biological triplicate with at least analytical duplicate analysis. Data analysis was conducted using JMP Pro 16 (Cary, NC, USA), and a full factorial design was analyzed to elucidate differences between protein type, percent addition, processing method. The three-way interaction was analyzed using Tukey’s HSD to determine significant differences between samples, with a *p*-value of less than 0.05 considered statistically significant.

## Figures and Tables

**Table 1:** Color of Concord grape gels with added 4, 6, and 8% pea or chickpea protein structured by HPP or heat.

Protein	Addition	Treatment	L*	a*	b*	ΔE Control ^1^	ΔE Process ^2^	BI
Pea	4%	Raw	35.3 ± 1.8 ^E^	4.9 ± 0.4 ^A^	−1.6 ± 0.5 ^E^	-	-	-
		Heat	35.5 ± 0.8 ^DE^	5.0 ± 0.2 ^A^	−1.1 ± 0.1 ^E^	0.5	-	6.8
		HPP	38.1 ± 2.2 ^ABCDE^	4.2 ± 0.8 ^A^	−1.9 ± 0.2 ^E^	3.0	3.4	2.9
	6%	Raw	37.2 ± 0.8 ^ABCDE^	4.7 ± 0.2 ^A^	−1.8 ± 0.2 ^E^	-	-	-
		Heat	36.9 ± 0.6 ^BCDE^	4.5 ± 0.2 ^A^	−1.4 ± 0.2 ^E^	0.5	-	4.8
		HPP	35.8 ± 1.3 ^CDE^	4.3 ± 0.7 ^A^	−2.1 ± 0.2 ^E^	1.5	1.6	2.8
	8%	Raw	38.9 ± 1.7 ^ABCE^	4.4 ± 0.2 ^A^	−1.7 ± 0.2 ^E^	-	-	-
		Heat	39.8 ± 1.2 ^AB^	3.6 ± 0.2 ^A^	−1.2 ± 0.2 ^E^	1.5	-	3.4
		HPP	37.1 ± 2.2 ^BCDE^	3.7 ± 0.7 ^A^	−2.2 ± 0.2 ^E^	2.2	3.7	1.3
Chickpea	4%	Raw	40.4 ± 0.9 ^AB^	4.5 ± 0.3 ^A^	0.8 ± 0.1 ^CD^	-	-	-
		Heat	38.4 ± 1.9 ^ABCDE^	4.6 ± 0.6 ^A^	1.8 ± 0.4 ^BC^	3.0	-	13.1
		HPP	36.8 ± 0.6 ^BCDE^	5.0 ± 0.2 ^A^	0.2 ± 0.2 ^D^	4.0	4.2	10.0
	6%	Raw	39.7 ± 0.9 ^ABC^	4.7 ± 0.4 ^A^	0.8 ± 0.3 ^CD^	-	-	-
		Heat	39.4 ± 0.8 ^ABCD^	4.1 ± 0.9 ^A^	2.7 ± 0.2 ^AB^	4.3	-	14.2
		HPP	38.1 ± 0.2 ^ABCDE^	4.7 ± 0.7 ^A^	0.3 ± 0.1 ^D^	1.9	7.2	9.4
	8%	Raw	41.1 ± 2.1 ^A^	3.9 ± 1.6 ^A^	2.1 ± 0.3 ^AB^	-	-	-
		Heat	39.5 ± 3.1 ^ABC^	3.6 ± 0.9 ^A^	3.1 ± 0.4 ^A^	2.6	-	14.4
		HPP	39.7 ± 1.6 ^ABC^	4.2 ± 0.8 ^A^	1.6 ± 0.2 ^BC^	1.7	2.9	11.4

^1^ Difference in color from corresponding raw sample. ^2^ Difference in color between heat and HPP. ^A–E^ Different letters are significantly different in the same column (*p* < 0.05).

**Table 2:** Nine-point hedonic scores for 6% pea protein-enriched whole Concord grape gel structured by HPP or heat.

Treatment	Appearance	Color	Aroma	Flavor	Texture	Overall
Heat	5.48 ± 1.76 ^A^	6.24 ± 1.55 ^A^	6.42 ± 5.11 ^A^	6.15 ± 1.61 ^A^	4.80 ± 2.01 ^A^	5.69 ± 1.68 ^A^
HPP	4.90 ± 1.67 ^B^	6.16 ± 1.44 ^A^	5.11 ± 1.55 ^B^	4.98 ± 1.82 ^B^	4.04 ± 1.84 ^B^	4.59 ± 1.80 ^B^

^A,B^ Different letters are significantly different for attribute (*p* < 0.05).

**Table 3:** Check All That Apply (CATA) for characteristics of fruit purees important to consumers.

Plant Protein	High in Protein	Minimally Processed	High in Antioxidants	High in Fiber	Clean/Short Ingredient Label	No Added Sugar
20%	55%	49%	42%	53%	55%	55%

**Table 4:** Nutritional composition of 6% protein-enriched whole Concord grape gels per 140 g serving size [2,41,42].

Protein Type	Crude Protein (g)	Crude Fiber (g)	Fat (g)	WSC (g)	Ash (g)
Pea	9.6	5.2	0.9	19.4	1.6
%DV	14% ^1^	19%	1%	7%	-
Faba	9.6	5.0	2.7	18.7	1.7
%DV	13% ^1^	18%	3%	7%	-

^1^ Protein %DV calculated by analytical determination of PDCAAS. WSC: Water soluble carbohydrate.

## Data Availability

The data that support the findings of this study are available from the corresponding author, O.I.P.-Z., upon reasonable request.
